# Human trafficking and exploitation: A global health concern

**DOI:** 10.1371/journal.pmed.1002437

**Published:** 2017-11-22

**Authors:** Cathy Zimmerman, Ligia Kiss

**Affiliations:** London School of Hygiene and Tropical Medicine, London, United Kingdon

## Abstract

In this collection review, Cathy Zimmerman and colleague introduce the *PLOS Medicine* Collection on Human Trafficking, Exploitation and Health, laying out the magnitude of the global trafficking problem and offering a public health policy framework to guide responses to trafficking.

**Summary points**

- Labor migration is an economic and social mobility strategy that benefits millions of people around the world, yet human trafficking and the exploitation of low-wage workers is pervasive.
- The negative health consequences of human trafficking—and labor exploitation more generally—are sufficiently prevalent and damaging that they comprise a public health problem of global magnitude.
- Human trafficking and labor exploitation are substantial health determinants that need to be treated as preventable, drawing on public health intervention approaches that target the underlying drivers of exploitation before the harm occurs.
- Exploitative practices are commonly sustained by business models that rely on disposable labor, labyrinthine supply chains, and usurious labor intermediaries alongside weakening labor governance and protections, and underpinned by deepening social and economic divisions.
- Initiatives to address human trafficking require targeted actions to prevent the drivers of exploitation across each stage of the labor migration cycle to stop the types of harm that can lead to generational cycles of disability and disenfranchisement.

## Introduction

While migration within and across national borders has been an economic and social mobility strategy that has benefited millions of people around the world, there is growing recognition that labor exploitation of migrant workers has become a problem of global proportions. Human trafficking and other forms of extreme exploitation, including forced labor and forced marriage, now collectively under the terminological umbrella “modern slavery,” are reported to affect an estimated 40.3 million people globally, with 29.4 million considered to be in situations of forced labor [1]. PLOS is launching a collection of essays and research articles on “Human Trafficking, Exploitation and Health” to increase awareness of the problem and to urge health and nonhealth professionals alike to engage in international and local responses to protect the health of individuals and populations affected by trafficking.

Human trafficking is a multidimensional human rights violation that centers on the act of exploitation. The United Nations defines trafficking in persons as “the recruitment, transportation, transfer, harbouring or receipt of persons, by means of the threat or use of force or other forms of coercion, of abduction, of fraud, of deception, of the abuse of power or of a position of vulnerability or of the giving or receiving of payments or benefits to achieve the consent of a person having control over another person, for the purpose of exploitation” [2]. The elements of coercion, exploitation, and harm link human trafficking with other forms of modern slavery, forced labor and forced marriage.

In this introduction to the Collection on Human Trafficking, Exploitation and Health, we describe the magnitude of the problem, discuss the complex characteristics of trafficking, indicate the harm and associated health burden of trafficking, and offer a public health policy framework to guide robust responses to trafficking. Ultimately, however, in this introductory paper, we assert that human trafficking is a global health concern. That is, the health consequences of human trafficking are so widespread and severe that it should be addressed as a public health problem of global magnitude. Furthermore, because human trafficking has pervasive global health implications, we propose that these abuses—and perhaps labor exploitation more generally—be treated as preventable.

### The dimensions of human trafficking and global health implications

Early discussions about trafficking in persons focused almost solely on sex trafficking of women and girls and drew primarily on law enforcement responses. But human trafficking is now understood more broadly to occur in a wide array of low- or no-wage hazardous labor. In fact, the contemporary amalgam of mobility and low-wage labor fosters many opportunities for labor exploitation. Men, women, and children are trafficked for various purposes, including domestic servitude, agricultural and plantation work, commercial fishing, textiles, factory labor, construction, mining, and forced sex work as well as bride trafficking and petty crime [3–5]. These types of abusive work situations are especially viable in low- and middle-income countries [6] where low-cost labor is in high demand and where informal and precarious employment proliferates and labor governance is weak [7, 8]. A substantial proportion of human trafficking occurs within the same country, although international trafficking has received greater global attention [6].

**Fig 1:**
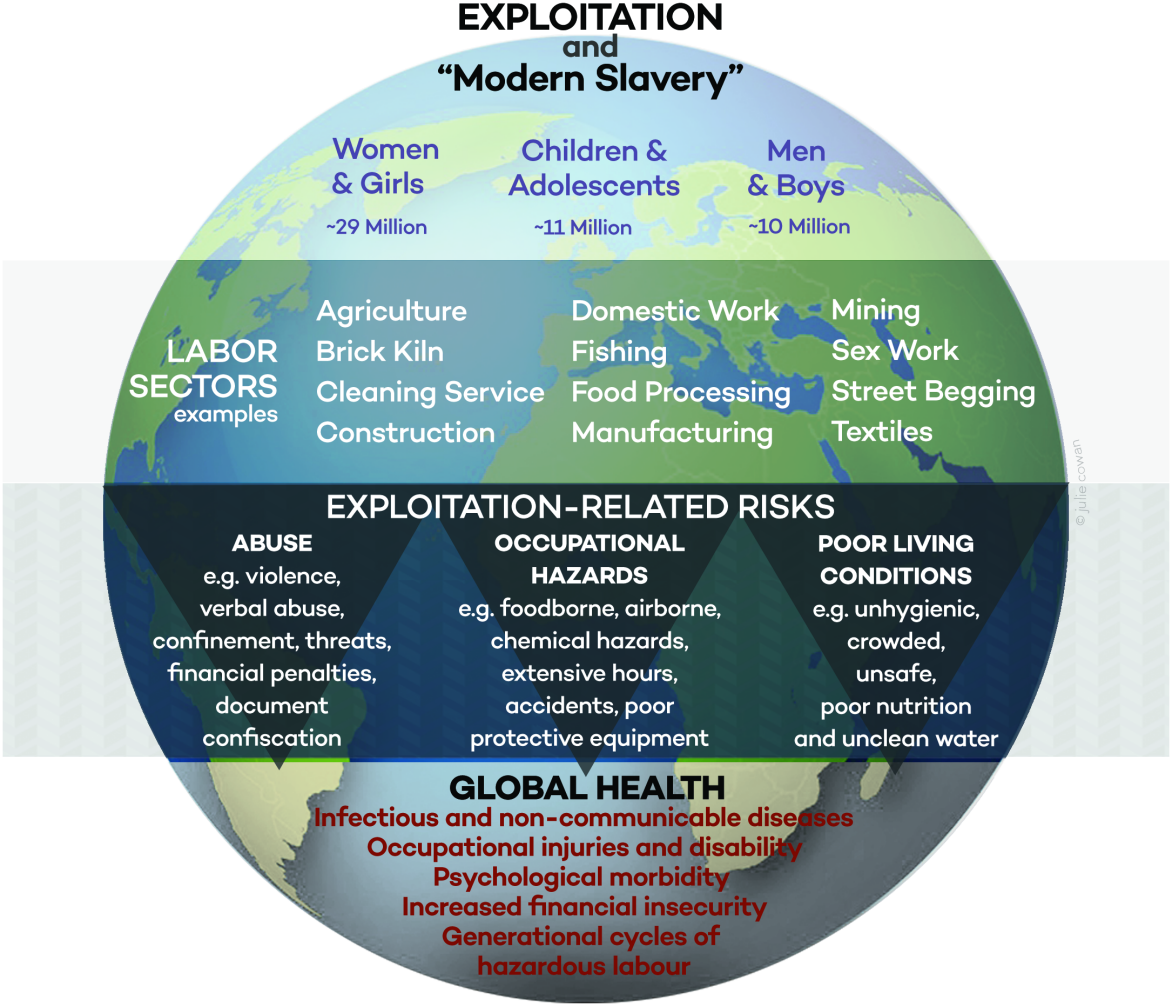
Exploitation, risks, and global health.

The exploitation that is at the heart of trafficking comprises different forms of abuse, such as extensive hours, poor pay, extortionate debt, physical confinement, serious occupational hazards, violence, and threats. These forms of abuse occur across a spectrum at varying levels of severity. And, importantly, the impact of exploitation on the health and wellbeing of a person who has been trafficked depends on the combination of types and severity of the acts she or he suffers (Fig 1).

### Harmful in what ways and to whom

There is growing evidence on the wide-ranging health consequences of human trafficking. A systematic review on health and human trafficking found that survivors experienced multiple forms of abuse, numerous sector-specific occupational hazards, and dangerous living conditions [9] and suffered a range of poor health consequences. Among trafficking surviors in Southeast Asia, nearly half (48%) reported physical or sexual abuse and 22% sustained severe injuries, including lost limbs, and reported symptoms indicative of depression and anxiety disorders [10]. At the same time, however, there has been limited evidence on the social, financial, and legal harm suffered by trafficked persons—which often have further implications for ill health.

Reports on human trafficking regularly highlight that child workers, minorities, and irregular migrants are at particular risk of more extreme forms of exploitation. Over half of the world’s 215 million young workers are estimated to be in hazardous sectors including forced sex work and forced street begging [11]. Ethnic minority and highly marginalized populations are known to work in some of the most exploitative and damaging sectors, such as leather tanning, mining, and stone quarry work [12]. Irregular or illegal migration status can be used to threaten and coerce workers. Poor language skills can prevent migrant workers from understanding and negotiating employment terms and enagaging in job training, and, importantly, it can hinder their understanding of local rights and assistance resources [13, 14]. Human trafficking also frequently manifests in highly gendered ways [1]. For example, women and girls are commonly trafficked for sexual exploitation, forced marriage, and domestic work [1, 4], while males appear to be more vulnerable to trafficking into various armed conflicts, and men in Southeast Asia are more likely than women to be recruited for commercial fishing, sometimes referred to as “sea slavery” [15, 16]. Government can play a role in restricting migration, such as Nepal’s migration bans affecting younger prospective female migrants [17], or can promote migration through, for example, the Bangladeshi government’s Memorandum of Understanding (MOU), which subsidizes recruitment fees for females migrating to numerous Gulf States [18].

### The public health burden of human trafficking and labor exploitation

Because of the challenges of conducting surveys on human trafficking, there has been little population-based prevalence data on trafficking-related morbidity and mortality. In fact, globally, there is very little research on the health of low-wage migrant workers in general, especially in low-income countries [19]. Nonetheless, broader research indicates that labor market inequalities are closely associated with mortality, healthy life expectancy, and injury rates [20, 21]. Takala et al. suggest there are 2.3 million work-attributable deaths annually, with the greater share of work-related morbidity and injuries in low-income countries, and highlight the gradual shift of hazardous labor to Asia, in particular [22]. The economic burden of work-related injury and illness on states is also substantial, with global estimates indicating a worldwide price tag of US$2.8 trillion [23]. While it is currently not possible to know how extreme forms of exploitation might be represented in such figures, especially in hazardous sectors in low- and middle-income countries, the probability that the health burden is substantial can hardly be discounted.

### Prevention: A public health approach

Recent epidemiological shifts away from infectious diseases towards noncommunicable diseases [24] has led to growing knowledge about the influence of socioeconomic and cultural determinants in mortality and morbidity patterns. This has resulted in increased recognition of the effect of precarious employment, multiple forms of marginalization, and legal and entitlement structures in individual and population health [14]. Addressing these structural determinants is at the core of effective prevention efforts for many public health problems. Extreme exploitation, like other complex social phenomena, such as violence against women or substance misuse, has multiple and interacting causes and effects [25, 26]. Labor exploitation can be seen as a health determinant and preventable social problem and benefit from public health prevention approaches that target the harm before it occurs [27]. A prevention lens directs us to consider the interaction of multiple factors that protect or put individuals and populations at risk of labor exploitation and to seek potential mechanisms to minimize these risks or enhance protection. It also suggests that we examine how various dimensions of exploitation might contribute to aspects of harm among different populations. Moreover, from this vantage point, we might reflect somewhat provocatively on the striking similarities between the harm sustained by people who are officially identified as “trafficking victims” versus migrant workers in the same sectors [19].

### A public health policy framework to address human trafficking, exploitation, and health

To prevent the exploitation of aspiring labor migrants, evidence is urgently needed on the determinants of exploitation and factors that promote safe migration and decent work. Moreover, theoretical or policy frameworks are required to look specifically at the ways that individual, group, and structural factors (including economic, social, legal, and policy-related aspects) influence exploitation and health along a migration trajectory, which can guide our search for evidence to inform interventions [28–31].

**Fig 2:**
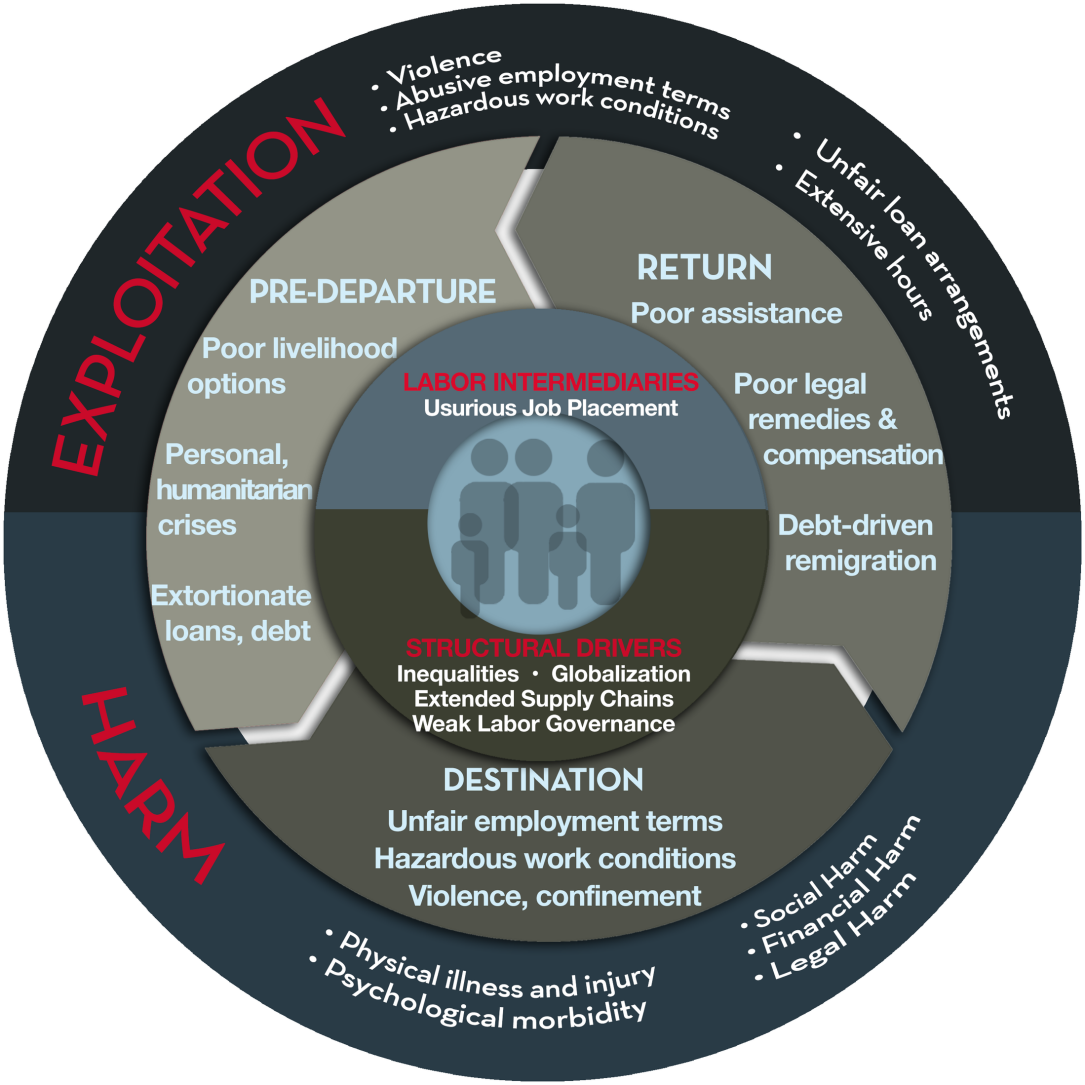
Socioeconomic determinants of labor exploitation and harm.

Fig 2 depicts factors associated with labor exploitation across a migration process, dimensions of exploitation, and various dimensions of harm. It is worth noting, however, that while structurally driven social, economic, and gendered power imbalances underpin exploitation more generally, they often manifest differently between different forms of exploitation. For example, there are critical distinctions between various types of labor trafficking and sex trafficking versus conflict-related trafficking. In many low-wage production sectors, for instance, exploitative practices are sustained by business models that rely on labyrinthine supply chains, myriad labor intermediaries, and high demand for inexpensive and disposable labor. It is not coincidental that exploitation of workers has occurred alongside the diminishing power and density of trade unions and shrinking freedom of association and collective bargaining [32]. These interactions are exacerbated by weak labor governance [33] that fails to protect workers from production processes frequently fueled by demands for low-cost goods and services—despite international conventions to protect workers [34]. The framework in Fig 2 depicts a process of complex, cumulative causation of potential harm throughout a migration cycle. It highlights interactions between macrolevel structural factors (e.g., global, national, social, etc., systems and institutions) that influence the persistence of trafficking and harm among individuals in communities (microlevels). And, while not explicit, this conceptualization also acknowledges the role of inequalities such as age, gender, nationality, ethnicity, and class [35] to each individual’s vulnerability to exploitation [36].

Labor intermediaries and migrant networks frequently play a key role in recruitment processes. Some labor recruiters may assist with job placement into decent work, while others might facilitate exploitation. Unscrupulous intermediaries are known to use extortion, deception, or coercion to exploit workers or to usher them towards abusive employers [37]. Notably, people can be recruited into trafficking situations multiple times over a single journey. Labor intermediaries can include a chain of connected or separate, formal or informal, trustworthy or untrustworthy agents. For instance, Nepali workers from rural areas often seek jobs abroad (e.g., domestic work, construction jobs) through a local agent who connects them to more formal manpower agencies in urban centers [38]. Informal migrant networks or social networks are often thought to confer greater protection from exploitation; however, this is not always the case [39]. Recent research indicated that Bolivian migrants were exploited by compatriots for textile work in Argentina, whereas the opposite was true among Kyrgyz construction workers who secured decent work in Kazakhstan through their own Kyrgyz networks [19]. Additionally, as recruitment processes or networks become more established, they can become a regular labor conduit, potentially feeding people into exploitative situations [40].

Importantly, this framework conceptualizes exploitation as a potentially preventable cause of harm [41]. This perspective incorporates forms of harm beyond physical, psychological, and occupational health problems and includes social, financial, and legal harm and further suggests that the damage from exploitation can transmit across generations.

The discussion that follows focuses primarily on trafficking of labor migrants and exploitation, but the core features underpinning exploitation, power, control, and abuse, are applicable to other forms of human trafficking (forced sex work, forced marriage, for armed conflict).

### Predeparture

Most migrants leave home in search of a better life for themselves and their family, sometimes inspired by income disparities between neighboring migrant and nonmigrant households. The effects of climate change on local production, market-driven land exhaustion, humanitarian crises, and weak social assistance have each contributed in different ways to distress migration [42]. Local livelihood challenges have pushed millions of individuals away from their homes towards income opportunities that are often difficult to refuse or in which conditions are nonnegotiable—including situations of human trafficking [43]. To reduce people’s vulnerability to extreme forms of exploitation, the international community has made substantial investments in community-based awareness raising and migration knowledge building [44]. These efforts are often based on the premise that, if individuals were more informed about migrating for work, they would be less susceptible to being exploited. However, there remains little evidence to demonstrate that human trafficking is caused by information deficits among prospective migrants or about the positive effects of premigration awareness interventions [45].

People may be at greater risk of entering potentially exploitative arrangements when they are compelled to make urgent migration decisions, such as when confronted by humanitarian crises such as armed conflict, environmental disasters (tsunamis, flooding, earthquake), organized and gang violence (e.g., Northern Triangle of Honduras, Guatemala, El Salvador), or personal crises such as family illness or death [46–48]. Household debt can push people to accept extortionate job placement or employment terms and conditions—and, conversely, people may take out loans at difficult repayment rates to fund their migration [49]. For example, 91% of Bangladeshi migrants reported multiple migration-related debts, including labor brokers’ fees [50]. Social support and job assistance schemes [51], where available, can mitigate distress migration but are sometimes perceived as inadequate to overcome financial pressures, long-term poverty, or to secure financial self-sufficiency [52].

### Destination

At the work destination, labor exploitation and related abuses and their converse, ‘decent, safe employment,’ are generally determined by a combination of employment arrangements and work conditions [28, 53]. The terms of employment set the parameters for the ways and extent to which a person can be exploited (e.g., low wages, piecework pay, extended hours, penalties for early termination of contract). For instance, among posttrafficking service users in the Mekong, an average work day (7 days per week) for fishermen was 19 hours, was 15 hours for domestic workers, and was 13 for factory workers [54]. Trafficked individuals are rarely given a contract, and if one is provided, they may not be able to read or change it [38]. Workers are rarely provided personal protective equipment (PPE) or medical insurance and few workplaces are equipped with health or safety measures, especially in less regulated sectors. Labor inspections are also uncommon, and when they do occur, inspectors are unlikely to check if workers are trafficked [55].

### Return

After being exploited, many trafficked workers are encumbered by physical and/or psychological health problems and debt. Trafficking victims seldom have access to health or social assistance or legal remedies such as financial compensation for work-related injuries or illness, disability-related lost future earnings, or unpaid wages. Debts and other financial obligations, including for medical care, can increase survivors’ vulnerability to further exploitation [49]. Additionally, returnee migrants who failed to gain the income they and their family expected commonly feel deep disappointment and sometimes stigma, which can lead to poor mental health outcomes and potential risk of retrafficking [56, 57]. Moreover, when one family member is disabled, other family members, including children, may be pushed into exploitative situations. This can begin a generational cycle of entry into hazardous labor, such as has been observed among families and children working in palm oil plantations in Indonesia, mica mines in India, or tobacco farms in the United States [58, 59].

Because there has been limited theoretical work conducted on labor exploitation and harm, this broad framework is meant to help guide future intervention research and prevention strategies. However, each of the categories and variables proposed must be understood within differing historical and socioeconomic contexts and the reigning political climate that might, for instance, fuel discriminatory public discourse on migrants and migrant workers.

## Discussion

Slavery and its like have existed for millennia; so have social and economic inequalities. Through the declaration of the 2030 Sustainable Development Goals, the international community has promised that efforts will be dedicated to reducing poverty, ensuring healthy lives, and, most encouragingly, promoting decent work. This brings us back to the proposition we posed initially: human trafficking should be considered a global health concern. First, in terms of prevalence, when compared with other well-recognised global health problems such as the approximately 35 million people infected with HIV or the 1 million girls under age 15 who give birth every year [60, 61], human trafficking seems to deserve similar attention, with current estimates at approximately 40.3 million people [1]. Next, when considering harm, findings from studies around the world indicate consistently that most trafficked people experience violence and hazardous, exhausting work, and few emerge without longer-term, sometimes disabling, physical and psychological damage [54].

To date, there has been very limited engagement by the global health community in the dialogue on or responses to trafficking. Similarly, those working to address “modern slavery” have given little attention to the health impact of trafficking. So, how does one bring these communities together? As the first medical journal collection on human trafficking, exploitation, and health, the *PLOS* collection offers a good start towards gaining greater attention from the health sector. Providing evidence alongside expert commentary, this collection points to the range of clinical specialties and policy considerations required to address human trafficking as a global health determinant. Similarly, initiatives to tackle modern slavery, forced labor, and human trafficking need to make the links between human trafficking and health by working more closely with the health sector [62]. For both communities, a public health approach that treats the harm from exploitation as preventable will help foster interventions on the large scale that is needed. We urgently need to know more about the health burden posed by exploitative, low-wage, and hazardous labor, and, most importantly, the associated risk factors, especially in Asia and Africa—locations where some of the most exploitative labor occurs [63]. This is the type of evidentiary groundwork that was laid to address complex social problems such as intimate partner violence and that is now included in many routine health surveys and the international calculation of the Global Burden of Disease [25, 64]. Importantly, to intervene in effective and efficient ways, evidence is also needed on the determinants of human trafficking and on who is most affected and in what ways so that precious funds for intervetions are well targeted. The ecological framework introduced in this paper might serve as a starting point to direct research to investigate key structural, social, and individual drivers of exploitation.

Moreover, a public health approach to prevent human trafficking should simultaneously generate greater attention to its less recognized sibling, labor exploitation. That is, initiatives to address human trafficking will benefit from including actions to prevent exploitation and harm among low-wage laborers, more broadly—in what is often known as 3D work: dirty, dangerous, and demeaning. A dialogue is needed about how much and in what ways low-wage workers are currently exploited and about the ways that work-related hazards might harm individuals, including by disabling parents, who may then be forced to send their children to work—perhaps producing a generational cycle of disability and disenfranchisement.

In an era in which the value of human labor appears to be systematically degraded and political rhetoric further marginalizes already disregarded migrants and disadvantaged workers, now is a propitious moment to launch, in earnest, global health actions to tackle endemic labor exploitation.

## Abbreviations

MOU: Memorandum of Understanding
PPE: personal protective equipment
